# Association between rheumatoid arthritis and risk of radiotherapy toxicity: a systematic review

**DOI:** 10.1136/bmjonc-2024-000407

**Published:** 2024-07-16

**Authors:** Nina Liebenberg, Alan McWilliam, Sarah L Kerns, Deborah C Marshall, Catharine M West

**Affiliations:** 1 Medicine, Royal Surrey County Hospital NHS Foundation Trust, Guildford, UK; 2 Division of Cancer Science, The University of Manchester, Manchester, UK; 3 The Christie NHS Foundation Trust, Manchester, UK; 4 Department of Radiation Oncology, Medical College of Wisconsin, Milwaukee, Wisconsin, USA; 5 Department of Radiation Oncology, Icahn School of Medicine at Mount Sinai, New York, New York, USA

**Keywords:** Radiotherapy

## Abstract

**Objective:**

There is sometimes concern over the use of radiotherapy for cancer in individuals with rheumatoid arthritis (RA), but there is little evidence to support its avoidance. Identifying any association between RA and risk of radiotherapy toxicity could impact current guidance. We aimed to review the evidence base.

**Design:**

Following Preferred Reporting Items for Systematic Reviews and Meta-Analyses 2020 guidelines, a systematic review was conducted of Medline, Embase and PubMed databases on 25 November 2019 and updated 22 February 2024. Articles identified for inclusion were reviewed by two independent assessors.

**Results:**

155 articles were identified. With repeat articles excluded, 114 remained. 12 articles were included in qualitative analysis. Six studies held no comparison cohort; one compared patients with RA to patients without RA collagen vascular disease (CVD); five compared patients with RA to CVD or a matched pair. Studies showed patients with RA developed higher levels of toxicity; however, only two studies had statistically significant results. Nine of the 12 studies had medium-to-low quality evidence and displayed predisposition to numerous biases. Due to limited high-quality research, it is difficult to draw a clear conclusion on the relationship between RA and radiotherapy toxicity.

**Conclusion:**

Given the current lack of strong and high-quality evidence identified in this review, dose reduction of radiotherapy in patients with RA lacks sufficient evidence to be recommended. There is a need for further high-quality research involving prospective analyses of toxicity, up-to-date radiotherapy techniques, long follow-up and large cohorts. Also, analyses need to adjust for confounding factors, match for risk factors and incorporate RA activity status assessments.

**WHAT IS ALREADY KNOWN ON THIS TOPIC**

- The management of patients with rheumatoid arthritis (RA) and cancer is varied and lacks guidance. A 2019 systematic review and meta-analysis of toxicity in patients with collagen vascular or inflammatory bowel disease (including those with RA) concluded that they were not contraindications for radiotherapy. A 2021 meta-analysis of case-control studies in patients with collagen vascular disease found those with RA had no increased acute toxicity, but a higher rate of late effects. Low absolute risks of severe toxicity were considered insufficient to justify treatment de-escalation.

**WHAT THIS STUDY ADDS**

- Our systematic review focused just on patients with RA. We drew the same conclusions as above, but also showed that most studies were of medium-to-low quality with limited internal validity and generalisability. We identified substantial heterogeneity between studies, making comparisons difficult and collective statistical evaluations prone to low clinical validity. Our study adds to the literature by highlighting the limitations of previous evidence and making recommendations for improving future research.

**HOW THIS STUDY MIGHT AFFECT RESEARCH, PRACTICE OR POLICY**

- We identified a need for further high-quality research in the area, which includes prospective toxicity collection, up-to-date radiotherapy techniques, larger cohorts of patients with RA, appropriate matching of risk factors, sufficient follow-up, incorporation of confounding factors in the analysis and assessment of RA disease activity. Use of real-world data should also be explored, along with better documentation of radiotherapy toxicities and RA severity in electronic health records.

## Introduction

 Over the last 50 years, survival rates have increased significantly for many cancers.^1^ A focus on not only treating malignancy but also minimising side-effects of therapy would improve the quality of life of the increasing population of cancer survivors.^2^ Radiotherapy is a key treatment for cancer, indicated for approximately 50% of patients.^3^ Toxicity occurring during treatment (acute) is generally reversible, but there can be long-term effects.^4 5^ Risk factors for developing toxicity include those related to radiation physics (dose, irradiated volume), tissue tolerance, use of concurrent chemotherapy, genetic predisposition and comorbidities.^6^,^12^ Identifying risk factors for individual patients could underpin tailored treatments.

Rheumatoid arthritis (RA) is a comorbidity that might affect risk of radiotherapy toxicity. RA is a systemic autoimmune disease involving persistent joint inflammation which results in cartilage and bone damage alongside systemic extra-articular effects.^13 14^ There is a potential link between RA and radiosensitivity.

Ionising radiations damage DNA and the most critical lesions are double-strand breaks (DSBs).^15^ Most DSBs are repaired by DNA damage response (DDR) pathways but are lethal if unrepaired.^15 16^ Genetic alterations in DDR genes can increase radiosensitivity as seen in several genetic syndromes, and they also play a role in the pathogenesis of RA.^12 17^ Polymorphisms in the many genes implicated in RA could result in a radiosensitive, immunodeficient phenotype, seen in previous literature with *ATM*.^1217^,^19^

There is also a potential link between RA and risk of radiotherapy toxicity via inflammatory processes that are involved in the aetiology of both RA and radiation-induced normal tissue injury.^20 21^ The systemic autoimmune and inflammatory nature of RA may suggest an increased likelihood of prolonged systemic inflammation and dysregulation of immune response following radiotherapy, amplifying normal tissue complications and predisposing an individual to toxicity.

There is no clear clinical guidance for patients with a comorbidity of RA undergoing radiotherapy. A theory of association is plausible due to the commonality of pathological pathways involved in RA and radiotherapy toxicity. Identifying any association between RA and radiosensitivity could therefore impact current clinical guidance and identify a focus for further research in this field. A 2019 systematic review and meta-analysis of toxicity in patients with collagen vascular or inflammatory bowel disease (including those with RA) concluded they were not contraindications for radiotherapy.^22^ A 2021 meta-analysis of case-control studies in patients with collagen vascular disease (CVD) found those with RA had no increased acute toxicity, but a higher rate of late effects. Low absolute risks of severe toxicity were considered insufficient to justify treatment de-escalation.^23^ As there has been none that focused specifically on RA, we aimed to conduct a systematic review to explore the relationship between RA and risk of radiotherapy toxicity. We also aimed to identify the limitations of previous work that could be addressed in future work.

## Methods

### Information sources and search strategy

Preferred Reporting Items for Systematic Reviews and Meta-Analyses (PRISMA) 2020 guidelines were followed.^24^ A literature search was completed using: ‘Titles’ and ‘Abstracts’ for Ovid Medline and Ovid Embase; and ‘Title/Abstracts’ for PubMed databases.

The following search terms were used:

(((((((toxic* OR side effect* OR adverse event* OR adverse effect*)) AND (radiotherapy OR radiat*)) AND (neoplasm* OR cancer* OR malignan* OR tumour* OR tumor*)) AND rheumatoid arthritis) AND (patient* OR cohort*)) NOT ultraviolet) NOT review

The search was initially completed on 25 November 2019 and then rerun and finalised on 22 February 2024 involving PubMed with no year limits; Ovid Embase 1974–2024 to 22 February 2024; Ovid Medline 1946 to 22 of February 2024 with one search run in the interim. Citations were reviewed in selected articles and systematic reviews found in the search. Citations found through additional reading were also included.

### Study selection

The inclusion criteria were as follows: participants of any age, treated with radiotherapy for any form of cancer; included patients with RA alone or as a subgroup of CVD; studies measured incidence of acute or late toxicity due to radiotherapy using toxicity based on outcome assessment tools (The Radiation Therapy Oncology Group (RTOG)/European organisation for Research and Treatment of Cancer (EORTC) or Common Terminology Criteria for Adverse Events (CTCAE)); studies were prospective or retrospective with a minimum of five patients with RA; no minimum time period reported.

The exclusion criteria were as follows: research studies of non-original research; review articles; case reports due to reporting bias; studies analysing CVD without a subgroup of RA; studies involving palliative radiotherapy only; studies investigating alternative forms of therapy as primary or secondary outcomes.

### Data collection

Once search terms were finalised and eligibility criteria identified, titles and abstracts were screened. A secondary reviewer assessed the abstracts identified for review and a consensus was agreed on the selection of articles requiring a full-text analysis and then for inclusion.

Data were organised into an Excel Spreadsheet which included the abstract or summary; cancer type; cohort size; radiotherapy regimen; toxicity scale; toxicity endpoint; finding; effect size; use of multivariable analysis.

A narrative review was conducted as data were unsuitable for a meta-analysis. A critical appraisal of the identified studies was carried out. Risk of bias and heterogeneity were narratively assessed.

The review and the protocol were not prospectively registered. No data from the review are currently publicly available.

### Quality assessment process

The quality assessment tool from Hawker *et al* was used.^25^ The tool contains nine questions ranking studies, respectively, between 1 and 4 from ‘very poor’ to ‘good’. A score of high quality (A) was described as between 30 and 36 points; medium quality (B) 24 and 29 points; low quality 9 and 24 (C) points.

## Results

### PRISMA and study characteristics

**Figure 1:**
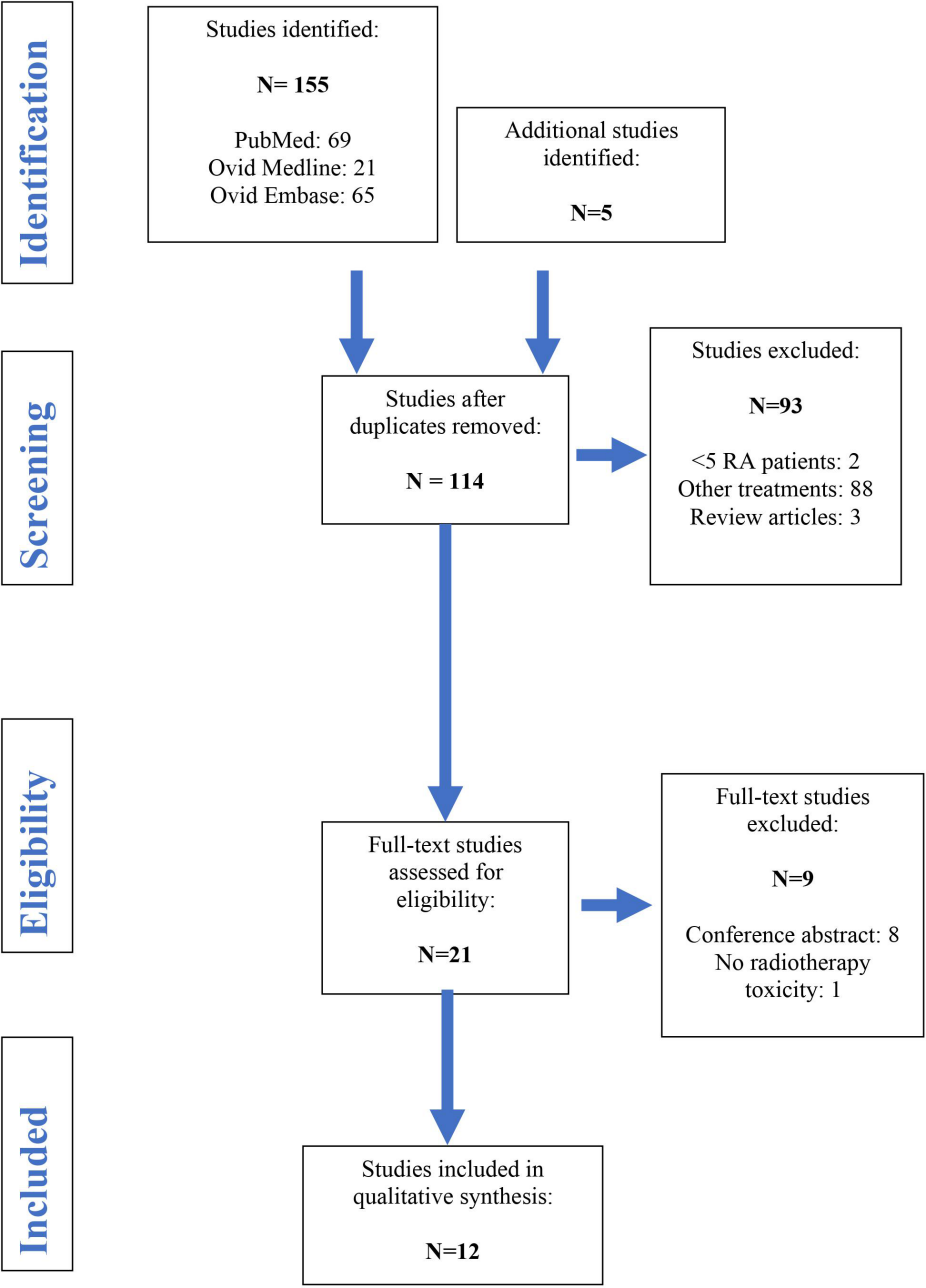
PRISMA flow chart describing data collection process.

Figure 1 shows the PRISMA flow chart. The search identified 155 studies. Five studies were added from citation searching and further reading resulting in 160 studies. Duplicates (n=46) were removed. Titles and abstracts of the remaining 114 studies were screened, excluding 93. The remaining 21 studies were assessed through full-text analysis. Eight studies were excluded as they were conference abstracts with insufficient information. One study was excluded due to lack of a radiotherapy toxicity endpoint. The final 12 studies were published between 1993 and 2021.

Tables1  2 outline key study characteristics of the 12 studies and results from meta-analyses present in the literature, published between 1993 and 2021.^2223 26^,^37^ Three studies held no comparison cohort; one compared patients with RA to patients without RA CVD; five compared patients with RA or CVD to a matched pair; three included multivariable analysis. Three studies were identified as high quality (grade A); seven studies as medium quality (grade B); and two as low quality (grade C) (table 3).^26^,^37^ Felefly *et al* showed low quality of methodology used, sampling, generalisability and implications.^26^ Chen *et al* demonstrated low quality of evidence with statistical abnormalities where the total number of patients investigated for late complications in the CVD and control group add to 39 and 78 respectively rather than the stated 36 and 72, with no explanation given.^28^

**Table 1:** Summary of study key characteristics for papers with single cancer sites

Study	Cancer	N	Radiotherapy	System	Toxicity	Finding	P value
Felefly *et al* 2017^26^	Gynae	11 RA	39.6–46 Gy EBRT (+10 Gy boost in 3 pts) + 15–20 Gy brachytherapy, 7 had concurrent chemo	CTCAE 4.0	Acute≥G3	1 pt	
					Late≥G3	1 pt	
Dong *et al* 2017^27^	Breast	40 RA 117 MP	46 Gy/2# +11/14 Gy boost 15 RA pts had concurrent chemo	CTCAE 4.0		RA versus MP	
					Acute≥G2	25.0% versus 13.7%. OR 2.1	0.08
					Late≥G2	7.5% versus 4.3% OR 1.8.	0.38
Chen *et al* 2001^28^	Breast	17 RA 4 SCL 4RP, 5 SLE 2 Sjogren’s 4 PLM, 72 MP	48 Gy/2# + 16 Gy boost*	RTOG EORTC		RA versus MP	
					Acute	12% versus 3%	0.21
					Late	10% versus 3%†	0.21
						CVD versus MP	
					Acute	14% versus 8%	0.40
					Late	17% versus 3%†	<0.05
Diao *et al* 2017^30^	Thoracic	19 RA 4 SLE 4SCL 3 Sjogren’s 1 mixed 825 controls	3D-CRT or IMRT dose >45 Gy Concurrent chemo in 15/25 patients with CVD	Comp		CVD versus MP	
					Esophagitis≥G3	22.6% versus 19.0%	0.64
					Pneumonitis≥G3	25.8% versus 9.9%	<0.05
					Pneumonitis≥G3	HR 2.92 (1.41, 6.03) MVA HR 2.19 (1.02, 4.72)	<0.05 <0.05
Lowell *et al* 2011^33^	Intracranial	8 RA 5 SLE 1 SCD 6 MS	GKRS 12–19 Gy	RTOG EORTC	Acute/Late≥G2	37.5% (3 out of 8 patients with RA)	
Yamomoto *et al* 2017^35^	Cervical	533 8 RA	30–41.4 Gy/1.8# EBRT +- 19–20 Gy/5–6# HDR-ICBT. Concurrent chemo in 363/533 pts*	Pelvic insufficiency fracture		MVA of patients with RA	
					Presence of fracture	HR 2.67 (1.07 to 6.62)	<0.05
Uezono *et al* 2013^36^	Cervical	99 5 RA	50.4 Gy/1.8# (45.0–50.4 Gy) EBRT +-central shielding dose 14.4 Gy (5.4–34.2) ±boost EBRT 4–8 Gy. 67 pts had concurrent chemo.	Pelvic insufficiency fracture		UVA of patients with RA	
					Presence of fracture	HR 1.26 (0.47 to 2.88)	0.62

* Adjuvant systemic therapy was administered as clinically indicated.

† Statistical abnormalities identified in the result claimed by this study.

AS, ankylosing spondylolysis; CF, conventional fractionation; CTCAE, Common Terminology Criteria for Adverse Events; 3D-CRT, 3 dimensional conformal radiation therapy; DLE, discoid lupus erythematosus; DM, dermatomyositis; EBRT, external beam radiotherapy; EORTC, European Organisation for Research and Treatment of Cancer; GKRS, gamma knife radiosurgery; HDR-ICBT, high-dose rate intracavitary brachytherapy; IMRT, intensity-modulated radiotherapy; j, juvenile; MCTD, mixed connective tissue disease; MH, moderate hypofractionation; MP, matched pair; MS, multiple sclerosis; MVA, multivariate analysis; PDM, polydermatomyositis; PF, per fraction; PM, polymyositis; RA, rheumatoid arthritis; RTOG, Radiation Therapy Oncology Group; SCL, scleroderma; SLE, systemic lupus erythematosus; UH, ultrahypofractionation; UVA, univariate analysis; WG, Wegener's granulomatosis.

**Table 2:** Summary of study key characteristics for papers with mixed cancer sites

Study	Cancer	N	Radiotherapy	System	Toxicity	Finding	P value
Fiorica *et al* 2021^37^	Mixed	29	82.8% CF: 1.8–2 Gy 17.2% HF: 5-8y. Total 20–40 Gy. Chemo 3/29.	CTCAE 3.0	Acute ≥G3 Late ≥G2	13.8% (4 out of 29 patients) 10.3% (3 out of 29 patients)	
Lin *et al* 2008^29^	Mixed	33 RA 13 SLE 9 DMM 5 AS 4 PMR 4 WG 3 MCTD 219 MP	Mixed radiotherapy Concurrent chemo in 18/86 CVD patients	RTOG EORTC		RA versus MP	
					Acute	64.9% versus 76.2%	
					Acute ≥G3	10.8% versus 9.9%	
					Late	29.7% versus 13.9%	
					Late ≥G3	2.7% versus 4.0%	
						CVD versus MP	
					Acute	65.1% versus 72.5%	
					Acute ≥G3	10.5% versus 10.4%	
					Late	29.1% versus 14.0%	<0.05
					Late ≥G3	9.3% versus 3.7%	0.08
Yoon *et al* 2021^34^	Mixed	74 RA 54 Psoriasis 34 SLE 8 SCL	CF: ≤ 2 Gy PF MH: >2 Gy to<5 Gy PF UH:≥5 Gy PF	CTCAE 5.0		MVA of patients with RA	
					Acute	OR 1.12	0.88
					Late	OR 2.56	0.15
Ross *et al* 1993^31^	Mixed	39 RA 13 SLE 4 SCL 4 DM 1 PM 61 MP	Mixed Concurrent chemo in 31/115 patients	RTOG		RA versus MP	
					Acute	14.2% versus 9.5%	
					Late	24.0% versus 5.0%	0.13
						CVD versus MP	
					Acute	11.4% versus 6.6%	0.38
					Late	9.8% versus 6.6%	0.69
					Fatal	5.0% versus 0%	
Morris and Powell 1997^32^	Mixed	131 RA 25 SLE 17 PDM/DM 8 AS, 5 jRA 3 DLE 4 MCTD	10–87.6 Gy±brachytherapy Concurrent chemo in 53/209 patients	RTOG/EORTC		RA versus non-RA CVD	
					Late	6.0% versus 21.0%	<0.05
Meta-analyses							
Lin *et al* 2019^22^	Mixed	Studies 6 Total 363 RA 296	No data collected	CTCAE	Acute grade ≥3	Weighted random-effect meta-analysis. Incidence (%)	
						8.7 (1.4 to 19.7)	
Shaikh *et al* 2021^23^	Mixed	Studies 5 Control 1116 RA 152	No data collected	CTCAE		Case control meta-analysis	
					Acute ≥G3 (Dong ≥G2)	OR 1.47 (0.84 to 2.56)	
		Studies 5 Control 1104 RA 137			Late ≥F3 (Dong ≥G2)	OR 2.56 (1.28 to 5.14)	

* Adjuvant systemic therapy was administered as clinically indicated.

† Statistical abnormalities identified in the result claimed by this study.

AS, ankylosing spondylolysis; CF, conventional fractionation; CTCAE, Common Terminology Criteria for Adverse Events; 3D-CRT, 3 dimensional conformal radiation therapy; DLE, discoid lupus erythematosus; DM, dermatomyositis; EBRT, external beam radiotherapy; EORTC, European Organisation for Research and Treatment of Cancer; GKRS, gamma knife radiosurgery; HDR-ICBT, high-dose rate intracavitary brachytherapy; IMRT, intensity modulated radiotherapy; j, juvenile; MCTD, mixed connective tissue disease; MH, moderate hypofractionation; MP, matched pair; MS, multiple sclerosis; MVA, multivariate analysis; PDM, polydermatomyositis; PF, per fraction; PM, polymyositis; RA, rheumatoid arthritis; RTOG, Radiation Therapy Oncology Group; SCL, scleroderma; SLE, systemic lupus erythematosus; UH, ultrahypofractionation; UVA, univariate analysis; WG, Wegener's granulomatosis.

**Table 3:** Quality of evidence

Study	Abstract/title	Intro/aims	Methods/data	Sampling	Analysis	Ethics/bias	Results	Generalisability	Implications	Total	Quality grade
Felefly *et al*^26^	4	3	1	2	3	2	4	2	2	23	C
Dong *et al*^27^	4	3	3	2	3	3	4	2	2	26	B
Chen *et al*^28^	4	3	3	2	2	2	2	2	2	23	C
Lin *et al*^29^	4	3	2	2	4	2	4	2	2	25	B
Diao *et al*^30^	4	3	3	2	4	2	4	2	3	27	B
Ross *et al*^31^	4	3	3	2	4	2	4	2	2	26	B
Morris and Powell^32^	4	3	3	2	3	2	3	2	2	24	B
Lowell *et al*^33^	4	3	2	2	3	2	4	2	2	24	B
Yoon *et al*^34^	4	3	4	4	4	4	4	3	3	33	A
Yamamoto *et al*^35^	4	3	4	3	4	2	4	4	3	31	A
Uezono *et al*^36^	4	3	4	2	4	3	4	4	3	31	A
Fiorica *et al*^37^	4	3	3	2	3	4	3	2	3	27	B

Quality of evidence was assessed using the quality assessment tool from Hawker *et al.*^25^

### Key findings

Collectively, the studies suggest that individuals with RA have increased radiotherapy toxicity but only two studies had statistically significant results.^32 35^ One of the studies was grade B on the Hawker *et al* Quality Assessment Tool and compared RA to non-RA CVD matched pairs.^32^ The second study was grade A on the Hawker *et al* Quality Assessment Tool and showed in multivariable analysis that patients with RA had a statistically significant increased risk of developing a late toxicity.^35^ Unlike other studies, Lin *et al* showed similar or greater toxicity (non-significant) in the matched pair of patients without RA.^29^ When investigating all patients with CVD compared with matched controls, radiotherapy toxicity was higher in the CVD cohort with statistical significance in three studies, yet subcohort analysis of RA when analysed in these cohorts had no statistical significance.^28^,^30^

### Critical analysis: sample size and study heterogeneity

Table 4 summarises the strengths and limitations of individual studies. Most studies involved large total sample sizes (>100) but low numbers of patients with RA. The low number of patients with RA and small effect sizes would impact on the lack of statistical significances. RA is a broad spectrum of disease with multiple genes implicated.^17 18^ Having low numbers of patients with RA in each study reduces generalisability for all patients with RA. The studies were additionally heterogeneous. Four studies analysed mixed cancers with limited information on radiotherapy doses and regimen.^29 31 32 34^ This heterogeneity reduces internal validity of the results as toxicity can vary dependent on both cancer and radiotherapy regimen even if pairs are matched.^6 7^ There was also variability in length of follow-up and inclusion of potential confounding factors.

**Table 4:** Strengths and limitations within studies

Study	Pts	Patients with RA	Cancer	Radiotherapy	Comparison	Statistical analysis	Median FU (months)	Treated
Felefly *et al*^26^	<100	11	Single	Varied	None	None	56	1990–2015
Dong *et al*^27^	>100	40	Single	Similar	Non-CVD MP	Univariable due to matching	94	1981–2016
Chen *et al*^28^	<100	17	Single	Similar	2:1 non-CVD MP	Yes	150	1975–1998
Lin *et al*^29^	>100	33	Mixed	Varied	Non-CVD MP	Univariable due to matching, no subgroup analysis of RA	16	1985–2005
Diao *et al*^30^	>100	19	Single	Similar	Non-CVD MP	Univariable and multivariable but no subgroup analysis of RA	55	1998–2014
Ross *et al*^31^	>100	39	Mixed	Varied	Non-CVD MP	Statistical analysis	CVD:16; MP:30	1966–1990
Morris and Powell^32^	>100	131	Mixed	Varied	Non-RA CVD MP	Statistical analysis	RA 40; MP 55	1970–1995
Lowell *et al*^33^	<100	8	Single	Similar	None	None	16	2004–2009
Yoon *et al*^34^	>100	74	Mixed	Varied	Not applicable	Univariable and multivariable	23	2007–2019
Yamamoto *et al*^35^	>100	8	Single	Similar	Not applicable	Univariable and multivariable	60	2003–2012
Uezono *et al*^36^	<100	5	Single	Similar	Not applicable	Univariable and multivariable	21	2003–2009
Fiorica *et al*^37^	>100	29	Mixed	Similar	None	None	12	2005–2015

Green = strength. Red = limitation.

CVD, collagen vascular disease group; FU, follow-up; MP, matched pair; RA, number of patients with rheumatoid arthritis.

### Critical analysis: matched pair analysis

Six out of 12 studies used matched pair analysis, which was a strength. However, three studies did not state use of statistical analysis of matching and inconsistencies were present within studies (table 4).^26^,^35^ Felefly *et al*, Lowell *et al* and Fiorica had small sample sizes of patients with RA and did not use matched pairs or carry out statistical analysis limiting generalisability of the results.^26 33 37^ Morris and Powell had matched pairs, but the comparison was between patients with RA and without RA CVD also reducing generalisability of the results.^32^ Matched pair analysis in Diao *et al* prior to treatment showed 9.7% of patients in the CVD group having pulmonary fibrosis compared with 1.2% in the non-CVD group (p=0.01).^30^ Fibrosis is a common late complication of radiotherapy therefore having a background of fibrosis prior to radiotherapy is likely to increase the effect size seen, influencing internal validity of results.^38^,^42^ The studies also had different lengths of follow-up, which in two studies differed between the patients with CVD and the matched pairs (table 4).^26^,^36^ Shorter follow-up times in the CVD group were reported by Ross *et al* and Morris and Powell. Shorter length of follow-up in one group may underestimate the effect size, reducing the validity of outcomes.

### Critical analysis: concurrent medication

Nine of the 12 studies reported on concurrent medication use. Four of the studies showed the medication—oral cytotoxic rheumatological agents, methotrexate and non-steroidal anti-inflammatory drugs—affected risk of radiotherapy toxicity (table 5).^26^,^36^ Uezono *et al* included chemotherapy in their multivariable analysis, however, did not include other forms of systemic treatments.^36^ Yoon *et al* included multiple systemic treatments however did not specify what they were.^34^ Fiorico *et al* identified the biological drugs affecting the number of patients that experienced flares of RA in the 12 months following radiotherapy.^37^ Lin *et al* highlighted that many medications apply only to patients with CVD therefore could not be matched or accounted for in the overall model.^29^ The lack of medication matching is an unavoidable limitation in matched pair studies.

**Table 5:** Antirheumatic medication reported in studies

Study	Medication	Comparison	Toxicity	Effect	P value
Felefly *et al*^26^	Methotrexate (n=5, stopped during RT in 4)	Not analysed			
	Hydroxychloroquine (n=2)				
	NSAID (n=2)				
	Corticosteroid (n=1),				
Dong *et al*^27^	DMARDs (n=23)	No DMARDs (n=17)	Acute ≥G2 Late ≥G2	ns ns	
Chen *et al*^28^	Not reported/analysed				
Lin *et al*^29^	Corticosteroids in patients with CVD (n=32)	No corticosteroids (n=54)	Any acute/late	ns	
	NSAIDs in patients with CVD (n=34)	No NSAIDs (n=52)	Any acute/late	ns	
	Oral cytotoxic rheumatological agents in patients with CVD (n=17)	No oral cytotoxic rheumatological agents (n=69)	Any acute	Decreased	0.026
Diao *et al*^30^	Methotrexate in patients with CVD	No methotrexate	Pneumonitis ≥G3	Increased	0.030
	Hydroxychloroquine, prednisone, leflunomide, etanercept	Not analysed			
Ross *et al*^31^	Not reported/analysed				
Morris & Powell^32^	NSAIDs at the time of irradiation in patients with CVD (n=86)	No NSAIDs (n=94)	Any late toxicity	Decreased	0.040
	Corticosteroids	Not analysed			
Lowell *et al*^33^	Not reported/analysed				
Yoon *et al*^34^	Any CVD medication (n=121)	Not analysed			
Yamamoto *et al*^35^	Not reported/analysed				
Uezono *et al*^36^	Not reported/analysed				
Fiorica *et al*^37^	Patients taking conventional DMARDs experiencing RA flare (n=6)	Patients taking conventional DMARDs experiencing RA flare (n=16)		ns	
	Patients taking conventional DMARDs experiencing RA flare (n=5)	Patients taking conventional DMARDs experiencing RA flare (n=4)		Increased	0.005

CVD, collagen vascular disease; DMARD, disease-modifying antirheumatic drug; n, number of patients; ns, non-significant; NSAID, non-steroidal anti-inflammatory drug; RT, radiotherapy.

### Critical analysis: other considerations

The studies found through the literature review included patients treated up to 60 years ago before the introduction of modern conformal radiotherapy techniques associated with lower toxicity profiles. Earlier studies may be less generalisable to current practice. Also, the studies were all retrospective which predisposes recall bias, and were level 4 or below on the evidence-based pyramid implying substantial risk of further bias.

Most studies included sufficient statistical analysis; however, Felefly *et al*, Lowell *et al* and Fiorico *et al* assessed toxicity with no comparison cohort, which lends only low validity to any interpretation.^26 33^ Yoon *et al*, Yamamoto *et al* and Uezono *et al* included univariable and multivariable analysis.^34^,^36^ By incorporating factors suggested to affect toxicity (eg, age, concurrent treatment, comorbidities, planning target volume and dose fractionation), results are more valuable and likely to show the real impact of RA on toxicity.

## Discussion

The studies identified in this systematic review demonstrate variable methodology and risk of numerous biases. Only one study showed a high-quality statistically significant result for an association between RA and radiotherapy toxicity. Given the lack of strong evidence identified in the current review, dose reduction of radiotherapy in patients with RA lacks sufficient evidence to be recommended.

Our findings agree in part with those reported in the 2019 and 2021 meta-analyses that included patients with CVD and subgroup analyses of patients with RA.^22 23^ That is, there is some evidence for an increase in toxicity, but the absolute level is too low to warrant changing practice guidelines. Both meta-analyses and our study highlight the need for further high-quality research. Lin *et al* reported an incidence of late ≥G3 toxicity in patients with RA of 8.7% (95% CI 1.4 to 19.7).^22^ Shaikh *et al* showed patients with RA had increased ≥G2 late toxicity compared with a case control group (OR=2.56; 95% CI 1.28 to 5.14).^23^ Taken in isolation, these studies suggest that patients with RA have an increased risk of radiotherapy toxicity. In comparison to the meta-analyses, our study focused on methodology within and between studies. We showed heterogeneity in total radiotherapy dose, toxicity outcome, concurrent chemotherapy, cancer treated and time-frame of data collection, as well as risk of confounding bias from varied control matching. In addition, Shaikh *et al* included the statistics from Chen *et al* which was identified as being of low quality of evidence and had incorrect statistical analysis which may have affected the results of the meta-analysis. The above limitations highlight the need to interpret meta-analyses on this topic with caution and identify areas that must be addressed in future research to generate results of high validity in this field.

Multiple studies had a limitation due to the lack of a reliable method to assess CVD status, with Dong *et al* using disease-modifying antirheumatic drug (DMARD) use as a surrogate.^27 29^ Felefly reported that the single patient in their study who presented with symptoms of RA during radiotherapy suffered severe long-term complications in the treatment field indicating the importance of considering disease status of RA. Fiorica *et al* used the Disease Activity Score on 28 joints (DAS28) to assess disease activity with further subgroup analysis using this.^37^ DAS28, the American College of Rheumatology improvement criteria, the simplified disease activity index (SDAI) and the clinical disease activity index (CDAI) are all tools which would reliably assess RA status in research.^43^ Incorporating activity status assessments into research could provide more specific details as to why or when toxicities occur. Furthermore, disease status could be assessed before the start of radiotherapy, with potential to be incorporated into a toxicity risk calculation model.

In current practice, the management of patients with RA and cancer is varied and lacks guidance. There are two examples of varied practice identified in this systematic review.^30 33^ Diao *et al* highlighted that less than half of the patients with CVD carried on taking prescribed CVD directed medication during cancer treatment.^30^ Lowell *et al* reported use of radiotherapy doses at the lower end of the recommended range in patients with CVD.^33^ A review article found that DMARDs should be used with caution; however, there is a lack of clear evidence.^44^ The same article showed recommendations for the treatment of patients with RA and cancer often fail to meet methodological criteria and concluded a need for further research in the management of cancer in patients with RA.^44^ Given the current lack of strong evidence identified in the current review, dose reduction of radiotherapy in patients with RA lacks sufficient evidence to be recommended due to a potential risk of reducing the probability of cure. All measures to reduce toxicity should however be maximally explored in the setting of possible predisposition to toxicity; especially in the setting of RA-related organ involvement, for example, interstitial lung disease as would be appropriate for patients with underlying comorbidities of singular organs.^45^ Exploring the relationship between RA and radiotherapy toxicity through further high-quality research is vital to understand if dose adaptation is required to maximise quality of life without compromising survival.

Use of real-world data (RWD) could address the current limitations of research identified in this review. There may be opportunity to use electronic health records (EHRs) linked cohorts such as QResearch, a database consisting of anonymised health records drawn a computer system used in primary care; UK Biobank and Genomics England’s 100 000 Genomes project which link data from primary and secondary care with additional information specific to the cohort; Cancerlinq which provides a cancer-specific data set. RWD can allow rapid data collection, which would keep up-to-date with the ever developing radiotherapy techniques.^46^ RWD also allow analysis of large sample sizes that could detect smaller increases in radiotherapy toxicity risk particularly in commonly under-represented comorbidities such as RA.^46^ In routine data collection, healthcare professional and patient-reported outcomes (PROs) of radiotherapy toxicity would enrich datasets further.^47^ Several scales are available, for example, CTCAE, Late Effects Normal Tissue Task Force—Subjective, Objective, Management, Analytic, RTOG/EORTC, EORTC Quality of Life Questionnaire and PRO-CTCAE.^48^,^50^ For RA, it would be important to identify the severity of RA using verified scales (DAS28, SDAI and CDAI) and to collect medication data to allow for either matching or adjustment based on the use of RA drugs. Given that much of the data in registries are drawn from EHRs, there is a need for consistent documenting of radiotherapy toxicity in routine clinical practice and appropriate coding. We acknowledge that it is not a simple task to translate EHR-based codes into phenotypes, but there are many successful examples of doing so.^51^ It would be relatively straightforward to identify RA cases. Radiotoxicities would be more challenging, but it has been done in cardiac toxicity following lung radiotherapy and we were recently able to do it with irradiation cystitis.^52 53^

Our review had some limitations. The study was not registered prospectively. In addition, we used broad inclusion criteria, which would account for the heterogeneity seen in the results. However, our broad criteria allowed us to maximise the number of studies identified for analysis offering a more thorough picture of the current state of the literature.

## Conclusions

There are plausible mechanisms by which patients with RA would be more sensitive to radiotherapy, but there is currently a lack of high-quality research on this topic. A literature search identified 12 studies that overall suggest patients with RA do develop higher rates of radiotherapy toxicity; however, most studies lacked statistical significance, had varied methodology, low generalisability and risk of numerous bias. Given the current lack of strong and high-quality evidence identified in this review, dose reduction of radiotherapy in patients with RA lacks sufficient evidence to be recommended. There is a need for further research that would provide a step towards addressing the lack of current guidance for patients with RA. The field requires prospective studies using up-to-date radiotherapy techniques, larger cohorts of patients with RA, appropriate matching of risk factors, sufficient follow-up and incorporation of confounding factors in the analysis and disease activity of RA. The use of RWD that exploit EHRs could also address the limitations identified in our review.

## Data Availability

All data generated or analysed during this study are included in this published article.
