# Correction: Association between rheumatoid arthritis and risk of radiotherapy toxicity: a systematic review

**DOI:** 10.1136/bmjonc-2024-000407corr1

**Published:** 2025-04-23

 Nina Liebenberg, Alan McWilliam, Sarah L Kerns, *et al.* West- Association between rheumatoid arthritis and risk of radiotherapy toxicity: a systematic review: *BMJ Oncology* 2024;3:e000407.

Article type has been changed from ‘Review’ to ‘Original Research’.

